# NO signaling and S-nitrosylation regulate PTEN inhibition in neurodegeneration

**DOI:** 10.1186/1750-1326-5-49

**Published:** 2010-11-10

**Authors:** Young-Don Kwak, Tao Ma, Shiyong Diao, Xue Zhang, Yaomin Chen, Janet Hsu, Stuart A Lipton, Eliezer Masliah, Huaxi Xu, Francesca-Fang Liao

**Affiliations:** 1 Department of Pharmacology, University of Tennessee Health Science Center, College of Medicine, 874 Union Avenue, Memphis TN, 38163, USA; 2 Del E. Webb Center for Neuroscience, Aging, and Stem Cell Research, Sanford-Burnham Medical Research Institute, 10190 North Torrey Pines Road, La Jolla, CA 92037, USA; 3 Department of Neurology, Wuxi the Second People’s Hospital, Affiliated to Nanjing Medical University, Jiangsu 214002 PR China; 4 Department of Neurosciences, University of California at San Diego, 9500 Gilman Drive, La Jolla, CA 92039, USA

## Abstract

**Background:**

The phosphatase PTEN governs the phosphoinositide 3-kinase (PI3K)/Akt signaling pathway which is arguably the most important pro-survival pathway in neurons. Recently, PTEN has also been implicated in multiple important CNS functions such as neuronal differentiation, plasticity, injury and drug addiction. It has been reported that loss of PTEN protein, accompanied by Akt activation, occurs under excitotoxic conditions (stroke) as well as in Alzheimer's (AD) brains. However the molecular signals and mechanism underlying PTEN loss are unknown.

**Results:**

In this study, we investigated redox regulation of PTEN, namely S-nitrosylation, a covalent modification of cysteine residues by nitric oxide (NO), and H_2_O_2_-mediated oxidation. We found that S-nitrosylation of PTEN was markedly elevated in brains in the early stages of AD (MCI). Surprisingly, there was no increase in the H_2_O_2_-mediated oxidation of PTEN, a modification common in cancer cell types, in the MCI/AD brains as compared to normal aged control. Using several cultured neuronal models, we further demonstrate that S-nitrosylation, in conjunction with NO-mediated enhanced ubiquitination, regulates both the lipid phosphatase activity and protein stability of PTEN. S-nitrosylation and oxidation occur on overlapping and distinct Cys residues of PTEN. The NO signal induces PTEN protein degradation via the ubiquitin-proteasome system (UPS) through NEDD4-1-mediated ubiquitination.

**Conclusion:**

This study demonstrates for the first time that NO-mediated redox regulation is the mechanism of PTEN protein degradation, which is distinguished from the H_2_O_2_-mediated PTEN oxidation, known to only inactivate the enzyme. This novel regulatory mechanism likely accounts for the PTEN loss observed in neurodegeneration such as in AD, in which NO plays a critical pathophysiological role.

## Background

PTEN, the phosphatase and tensin homologue deleted on chromosome 10, is one of the most frequently mutated tumor suppressors in human cancers. The major, and best characterized, function of PTEN is its lipid phosphatase activity which dephosphorylates PIP3 to generate PIP2, and thus antagonizes the PI3K activity in the activation of Akt [1,2]. PTEN is expressed in almost all types of neurons [3] and is critical in multiple CNS functions such as neuronal differentiation and synaptogenesis [3-5], neuronal plasticity [6], neuronal injury (e.g., axonal branching/regeneration) [7,8] myelin thickness of periphery nerves [9], and in drug addiction [10]. Our research has focused on elucidating novel roles for PTEN in neuronal death and neurodegeneration. We and others have reported that PTEN protein levels are reduced in AD brains, accompanied by elevated Akt phosphorylation [11-13]. We hypothesize that loss of PTEN protein is a key event regulating the PI3-K/Akt signaling, arguably the most important pro-survival pathway in neurons. In this study, we aimed to investigate the underlying molecular mechanism of PTEN loss.

Studies conducted in experimental models for cancer and diabetes have shown that PTEN regulation is rather complex. Multiple mechanisms might be involved in a decrease or loss of PTEN function, in addition to gene mutation and deletion. These mechanisms may include transcription and post-translational modifications (PTMs) which include phosphorylation, acetylation, oxidation and ubiquitination [14]. PTEN is a relatively stable protein but its stability can be reduced in certain conditions, such as zinc treatment in neurons [15]. Phosphorylation at the S/T380-385 and T366/S370 sites influences PTEN stability as well as negatively regulating its enzymatic activity [16,17]. Besides phosphorylation, the ubiquitin-mediated proteasomal pathway is also an important mechanism regulating PTEN protein stability. We recently identified NEDD4-1 as the first ubiquitin ligase (E3) for PTEN that regulates PTEN degradation in multiple cancer types and in neurons [15,18].

PTEN can be acutely regulated by oxidative stress and by endogenously produced reactive oxygen species (ROS) [19]. Oxidation of the active site cysteine residue(s) by ROS has long been recognized as a common mechanism regulating several key members of the protein tyrosine phosphatases (PTPs) including PTEN. A number of ROS species, including hydrogen peroxide (H_2_O_2_), superoxide, peroxynitrite and nitrosothiol, modify PTEN on the critical cysteine residue (C124) and inactivate its lipid phosphatase activity in multiple cancer cell lines [20-24]. To investigate the molecular mechanism(s) underlying PTEN loss in the brains of AD patients, we examined these oxidative events with a special focus on H_2_O_2 _and NO-mediated S-nitrosylation; the later, a process of reversible addition of NO to Cys-sulfur in proteins, has emerged as a major regulatory mechanism in fine-tuning many critical molecules in the neuronal death pathway and neurodegeneration [24]. To our surprise, only NO-mediated events lead to PTEN protein degradation, though both modifications inactivate PTEN's lipid phosphatase activity in neurons. To our knowledge, this is the first report of NO being the upstream signal that leads to a series of PTMs regulating PTEN protein degradation.

## Results

### S-nitrosylated PTEN levels are increased in MCI/AD brains, correlating with reduced PTEN and elevated P-Akt

Initially, we sought to investigate whether S-nitrosylated PTEN (SNO-PTEN) is produced in neurodegenerative disorders associated with high levels of nitrosative stress such as stroke, AD and Parkinson's disease (PD). We included in our tests those specimens taken from autopsy patients diagnosed at an early stage of AD, called mild cognitive impairment (MCI), and compared them to aged matched control brain specimens (i.e., patients died from disorders not related to CNS). The patient cohort and information are summarized in the table [Additional file 1].

**Figure 1:**
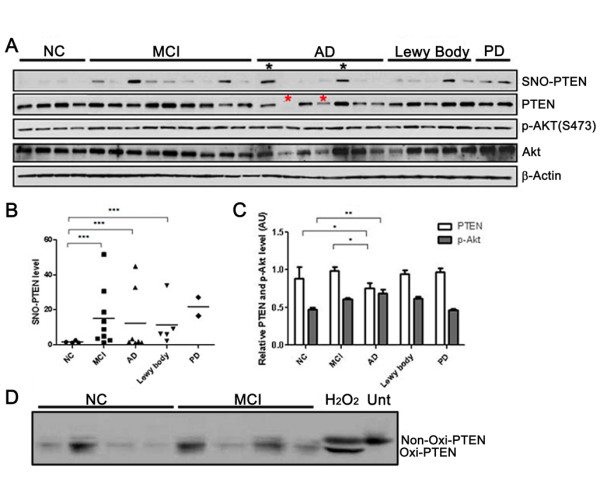
**Quantitative analysis of PTEN and P-Akt levels in relation to SNO-PTEN in human brains**. (A) SNO-PTEN levels were detected by immunoblot analysis following biotin-switch assays. Other proteins were detected by Western blot analysis using 15 μg of brain lysate. (B) Quantification of the Western blots using densitometry analysis reveals a statistically significant elevation of SNO-PTEN levels in MCI and AD brains compared to NC brains *** indicates *P *< 0.001. (C) An inverse correlation between PTEN and p-Akt levels. * indicates P < 0.05 in MCI and AD compared to NC brains for total PTEN levels. ** indicates P < 0.005 between AD and NC groups for P-Akt. (D) Examination of H_2_O_2_-type oxidation of PTEN by band-shift assays: neuronal cells or brains were lysed in buffer containing 2% SDS and 40 mM N-ethylmaleimide and 20 μg of proteins was subjected to 10% SDS-PAGE under non-reducing condition as described [22]. "Unt" stands for untreated neurons. H_2_O_2 _was used at 100 μM for 2 h as a positive control for band-shift of the oxidized PTEN.

From semi-quantitative profiling of PTEN/Akt in 27 human brains, we found that SNO-PTEN was markedly induced in the entorhinal cortices, the most vulnerable region in AD brains, as early as the MCI stage but was nearly undetectable in age-matched normal brains (NC) (Figure 1A). Although the number of specimens with matching ages is too small to conduct serious statistical analysis, SNO-PTEN was elevated in all the Lewy body and PD brains as examined by biotin-switch assays, suggesting nitrosylation of PTEN is a common denominator in these neurodegenerative disorders. Due to the sporadic nature of AD, it is not surprising that the level of SNO-PTEN is not inversely correlated with the level of PTEN protein in each individual case. However, statistical analysis reveals a trend (Figure 1B). Most significantly, the P-Akt was increased in the MCI samples compared to NC (Figure 1C) which correlates better with the elevated SNO-PTEN but not with the reduced PTEN protein in AD stage. Further analysis using Pearson's correlation analysis method revealed that SNO-PTEN is highly positively correlated with p-Akt level (0.8992) while PTEN level is negatively correlated with p-Akt (-0.6387) in AD samples. The inverse correlation between PTEN protein levels and P-Akt (Ser473) appears to be more significant for AD/NC than for MCI/NC, suggesting that SNO-PTEN may not cause immediate protein degradation. However, the great variation of total Akt levels among the AD samples makes it difficult for a solid conclusion. Interestingly, the two AD cases (indicated by black asterisks) showing the highest levels of SNO-PTEN were also affected by cerebral infarcts (strokes); the two AD cases marked by red asterisks showed very low levels or complete loss of PTEN and low levels of Akt protein but relatively high levels of phosphorylated P-Akt. Surprisingly, we did not observe a significant change in the oxidative status of PTEN between MCI and NC sample groups (Figure 1D), as determined by a band-shift assay on non-reducing gels as described [22,23].

### PTEN can be S-nitrosylated in cultured neuronal cells by exogenously and endogenously generated NO

**Figure 2:**
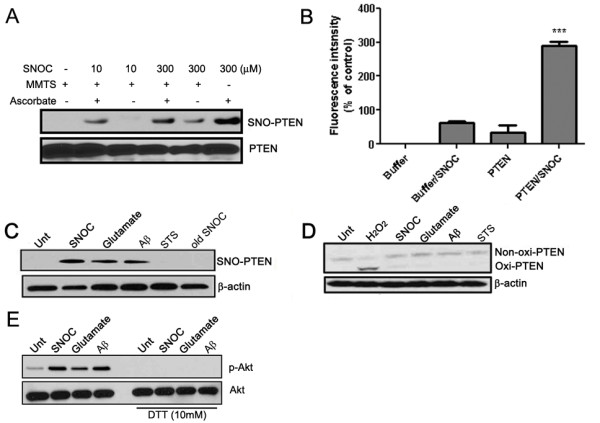
**PTEN is S-nitrosylated by various chemical and biological NO donors in cultured neurons**. (A) PTEN nitrosylation by SNOC in a dose-dependent manner as detected by biotin-switch assays: to detect S-nitrosylated cystein residues, the cysteine residues of PTEN was first masked by methylthiolation with MMTS. Nitrosothiols were then selectively reduced by ascorbate to reform free thiol group, which reacted with biotin-HPDH. In this experiment MMTS was added to serve as a positive control since all the cysteine residues in PTEN can react with biotin-HPDP. On the contrary, ascorbte in the untreated samples were used as negative control due to no reactive cysteine residues to biotin-HPDH. (B) Specificity of PTEN S-nitrosylation by DAN assay. (C) PTEN can be S-nitrosylated in cultured neurons by SNOC (200 μM, 30 min), glutamate (200 μM, 30 min), Aβ peptides (10 μM, 4 h) but not by staurosporine (STS, 200 nM, 30 min). (D) H_2_O_2_-induced oxidation in primary neurons with the same treatment conditions as in (C). H_2_O_2 _was used at 100 μM for 2 h. (E) P-Akt was detected by Western blot analysis 30 min after treatments with SNOC (200 μM), glutamate (200 μM), Aβ peptides (10 μM) in neurons. For the right panel, 10 mM DTT was added during the 30 min treatments.

Using cultured primary rat cortical neurons and biotin-switch assays, we found that SNO-PTEN can be rapidly induced in primary cultured cortical neurons treated with the physiological NO donor S-nitrosocysteine (SNOC) in a dose-dependent manner with detectable nitrosylation achieved by as little as 10 μM SNOC and a plateau with 300 μM (Figure 2A). This was also confirmed by a more quantitative fluorescent assay (DAN assay, Ref. [25]) using purified recombinant PTEN (Figure 2B). Additional neurotoxic compounds that induce NO generation, such as glutamate or β-amyloid peptides (Aβ_25-35_) also induced robust SNO-PTEN within minutes (Figure 2C) and lasted for more than 10 hours, even after the NO donors were removed from the cultured media. Interestingly, the classic apoptotic stimuli, staurosporine/STS, did not induce SNO-PTEN. Since STS is known to activate Ca^2+ ^influx through non-NMDAR type ion channels, our results indicate that the source of NO induced by glutamate/Aβ is most likely via the activated nNOS in response to NMDAR-mediated Ca^2+ ^influx. Two other mitochondrial ROS agents (rotenone and MPP^+^) also induced SNO-PTEN (data not shown); this is consistent with SNO-PTEN in PD brains and suggests that SNO-PTEN may play a common role in chronic degenerative diseases such as AD and PD. In parallel, we found that after treatment of SNOC/glutamate/Aβ, the majority of PTEN in neuronal cells (>85%) remained unmodified by H_2_O_2_-type oxidation, indicating that H_2_O_2 _may not be the dominant oxidizing species induced by these treatments (Figure 2D). This is consistent with one recent report [26]. Furthermore, we found that the same experimental conditions that induced PTEN nitrosylation led to Akt activation as assessed by increased P-Akt; both were diminished by DTT treatment (Figure 2E).

### NO signal induces subsequent enhanced ubiquitination of PTEN, leading to protein degradation via UPS

**Figure 3:**
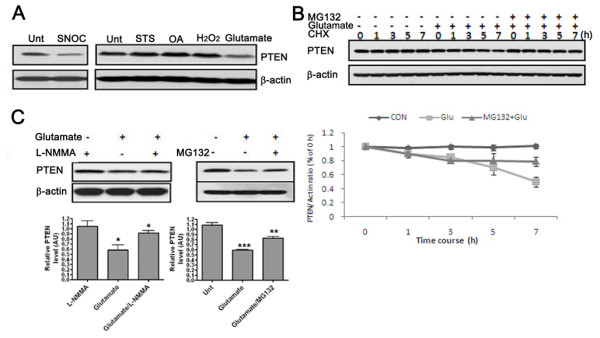
**PTEN S-nitrosylation correlates with protein degradation**. (A) Effects of various neurotoxins on the steady-state protein level of PTEN 4 h after treatments. N = 6 experiments. (B) Cycloheximide-chase experiment on PTEN stability. PRCN cells were incubated with 50 μg/ml cycloheximide (CHX) for the indicated times in the presence or absence of glutamate (200 μM). Cells were simultaneously treated with glutamate and cycloheximide. Two groups of neurons were also co-treated with glutamate and 25 μM MG132. Cells lysates were then prepared for Western blot analysis of steady-state levels of PTEN. (C) Glutamate-induced reduction of PTEN can be rescued by l-NMMA (1 mM) and MG132 (25 μM) pretreatment for 5 and 1 h, respectively, as examined by three independent experiments.

We found reduced PTEN (>50%) steady-state levels in neurons 2-4 h after exposure to SNOC, glutamate or Aβ_*25-35 *_peptides in a time-dependent manner (Figure 3A and 3B). Interestingly, the reduction in PTEN was not observed after H_2_O_2_, staurosporine (STS), or okadaic acid (OA). The strong correlation between SNO-PTEN formation and the reduced PTEN protein level suggests that the PTEN decrease may be a direct consequence of PTEN S-nitrosylation or may be caused otherwise by NO signaling. Indeed, pretreatment with a NOS inhibitor (l-NMMA) or the proteasome inhibitor MG132 for 5 h prior to glutamate exposure rescued the decrease in PTEN by over 70%and 50%, respectively (Figure 3C), suggesting protein degradation via the ubiquitin-proteasome system (UPS).

**Figure 4:**
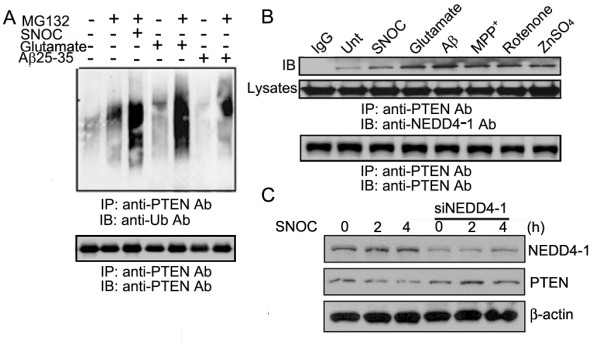
**NO signals induce enhanced ubiquitination of PTEN, leading to protein degradation**. (A) Treatments with SNOC, glutamate or Aβ peptides increase PTEN ubiquitination, as determined by IP-Western analysis. (B) Enhanced physical interaction between PTEN and NEDD4-1 upon various treatments at the conditions used in other experiments, as determined by co-immunoprecipitation/Western blot analysis. Figure is chosen as the representative of three independent experiments. (C) Downregulation of NEDD4-1 by siRNA ( 4 μg) prevents PTEN protein degradation upon SNOC treatment.

We also found that SNOC/glutamate/Aβ all induce enhanced PTEN ubiquitination with the maximum effect seen 2 h after treatments (Figure 4A). Consistent with enhanced ubiquitination, we found increased physical interaction between PTEN and its E3 ligase NEDD4-1 after these treatments (Figure 4B), as we published recently [15]. Moreover, downregulation of NEDD4-1 by its specific siRNA prevented PTEN degradation induced by SNOC treatment (Figure 4C). Together with the MG132 data, these findings indicate the involvement of the UPS in PTEN protein degradation. It is not clear whether S-nitrosylation of PTEN itself can lead to enhanced PTEN ubiquitination and protein degradation.

### SNO-PTEN likely occurs at multiple Cys residues in the phosphatase domain of PTEN, inactivating its lipid phosphatase activity

**Figure 5:**
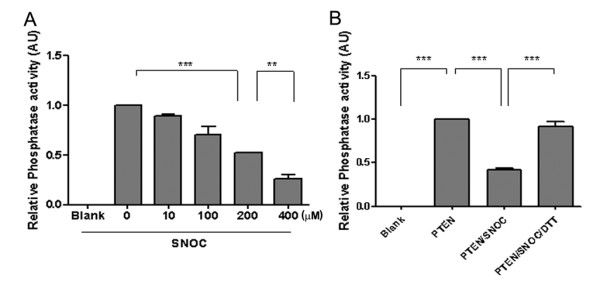
**SNOC treatment inactivates PTEN's lipid phosphatase activity**. (A) Dose-dependent effect of SNOC on lipid phosphatase activity as determined by Malachite Green assay. Data presented are means based on five independent experiments. ** indicates p < 0.05 and *** indicates p < 0.005. (B) DTT treatment (10 mM) completely restores the PTEN's lipid phosphatase activity abolished by SNOC (300 μM).

**Figure 6:**
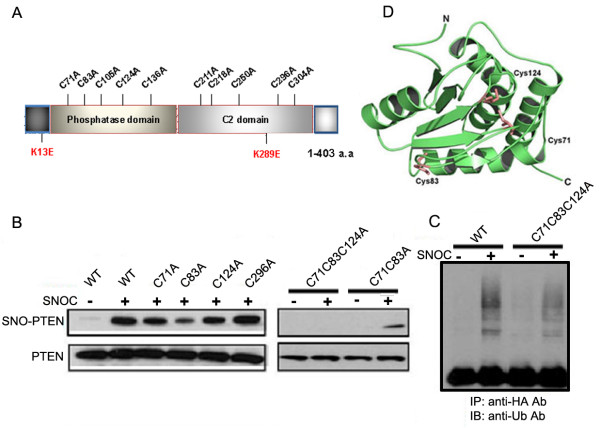
**Mapping the SNO-PTEN sites by site-directed mutagenesis**. (A) (Top scheme) Structural domains of PTEN: phosphatase domain, Ca-independent C2 and a PDZ binding domain at the C-terminus. (B) SNO-PTEN levels of Cys to Ala mutants as measured by biotin switch assays. Transfected N2a cells were treated with 200 μM SNOC for 30 min before cells were harvested for biochemical analysis. Data presented here is representative of 7 experiments. (C) PTEN ubiquitination as measured by IP-Western. Transfected WT or triple mutant PTEN were IPed with anti-HA antibody and immunoprobed with an antibody against ubiquitin. (D) Protein structure of the lipid phosphatase domain with three putative Cys residues labeled based on published PTEN crystal data [32].

We next examined the effect of PTEN S-nitrosylation on the lipid phosphatase activity of PTEN. By conventional Malachite Green assay using PIP3 as substrate [2], SNOC treatment inactivated PTEN in a dose-dependent manner with 80% of activity lost at 400 μM, which was reversed by DTT (Figure 5). To determine the target site(s) of SNOC on PTEN, we performed site-directed mutagenesis on each of the 10 cysteine residues (Cys to Ala replacement) (Figure 6A). Using biotin-switch assays, we examined the nitrosylation levels of each PTEN mutant after transfection into mouse neuroblastoma N2a cells 30 min after exposure to 200 μM SNOC and compared SNO-PTEN levels to those of WT PTEN. For the 10 PTEN mutants, only Cys83A mutant displayed significantly reduced S-nitrosylation [75% reduction based on the mean values of 7 experiments, Figure 6B and also see additional file 2]. Thus, the most likely candidate cysteine residue that is physiologically S-nitrosylated is C83. The C71A and C124A mutations only partially affected S-nitrosylation (~25%), and the double mutant C71C83A was nitrosylated similarly to the C83A mutant. The C71C124A double mutant had the same effect as single mutants. In contrast, triple mutant C71C83C124A completely abolished the PTEN nitrosylation, suggesting an involvement of all three Cys residues to varying degrees under our conditions. Furthermore, we also observed greatly reduced ubiquitination for the triple mutant (Figure 6C) which suggests the possibility that its ubiquitination may be partially dependent on PTEN nitrosylation. Interestingly, the steady-state levels of the triple mutant was not reduced by SNOC treatment as compared to WT or C71C83 double mutant [see additional file 3], indicating that the C124 residue is a critical determinant for PTEN protein stability.

### Downregulation of PTEN and elevated P-Akt levels protect neurons against Aβ-induced toxicity

**Figure.7:**
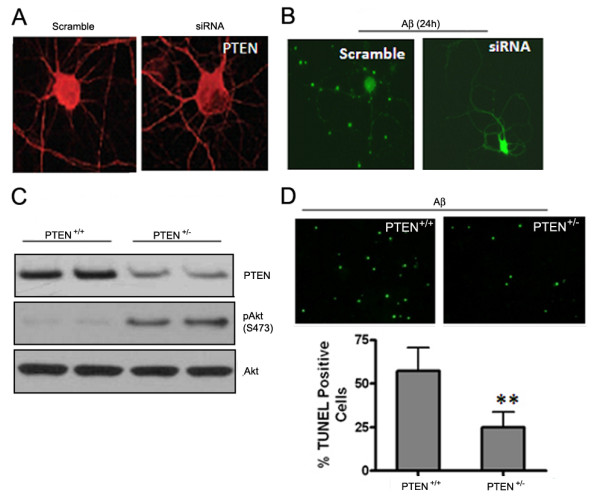
**Down regulation of PTEN is neuroprotective in acute experimental models**. (A) and (B) Downregulation of PTEN with specific siRNA confers neuroprotection 24 h after Aβ exposure (25 μM): PTEN IHC staining (red, A) and MAP/NeuN staining for neuron morphology (green, B). (C) Reduced PTEN protein level in PTEN heterozygous mouse brain (frontal region), accompanied by elevated P-Akt level (2-month-old mice, *n *= 2). (D) Primary cultured cortical neurons from PTEN +/- pups manifest less apoptotic cell death than PTEN+/+ neurons 24 h after Aβ exposure. Data presented as means ± SD from 5 independent experiments.

In cultured primary neurons, exposure to Aβ or NMDA induced a rapid decrease in PTEN and increase in P-Akt levels. Downregulation of endogenous PTEN via specific siRNA produces neuroprotection, as evidenced by preserved neuronal structures (Figure 7A and 7B; MAP2/NeuN staining/Green). Moreover, the frontal region of the brain of PTEN heterozygous mice bear reduced PTEN as compared to WT littermate controls (~50% reduction) and increased basal P-Akt levels (Figure 7C). Cortical neurons from PTEN+/- brains exhibited increased resistance to Aβ-induced cell death compared to those from WT littermate controls (Figure 7D), suggesting a neuroprotective role for PTEN downregulation.

## Discussion

PI3K/Akt is arguably the most important cell-survival signaling pathway for neurons. As the key negative regulator of the PI3K/Akt pathway, PTEN is an important target of study for neuroprotection during neurodegeneration. In this work, we are the first to demonstrate NO-mediated redox regulation as the mechanism of PTEN protein degradation. We also demonstrate that NO rapidly induces S-nitrosylation of PTEN, thereby inactivating it. Moreover, NO, but not H_2_O_2, _induces PTEN protein degradation. Loss of PTEN protein is reportedly associated with myocardial and brain ischemia [27-29], presumably as a cellular adaptive stress response to activate the pro-survival PI3K/Akt signaling. Herein, we show that PTEN loss also occurs in neurons in response to a variety of neurotoxins (e.g., glutamate and Aβ peptides) as well as in chronic neurodegenerative conditions such as AD and PD brains. Hence, our findings on NO-mediated PTEN protein degradation may represent a common mechanism underlying PTEN loss in these acute and chronic degenerative conditions, in which NO plays a critical pathophysiological role.

It is widely accepted that oxidative stress is one of the earliest changes that occurs in the pathogenesis of AD, arising from the imbalance between increased production of reactive oxygen and nitrogen species and impaired antioxidant defenses, as reflected in the accumulation of oxidative damage to macromolecules detected in MCI, the clinical precursor of AD, and AD brains [30,31]. H_2_O_2_-induced modification and S-nitrosylation represent the two dominant oxidative events through targeted modifications of critical Cys residues in proteins. H_2_O_2_-induced PTEN oxidation was reported to cause the formation of an intra-chain disulfide bond between C71-124 [22], and is reflected in faster mobility species by band-shift assay on non-reducing gels (Figure 2D). SNOC did not induce a band shift of PTEN on non-denaturing gels and thus unlikely induced a major conformational change due to intra-chain disulfide bond formation. However, our mutagenesis data suggest the involvement of overlapping residues on C71 and C124. It is, therefore, reasonable to predict that these two oxidative modifications can compete with each other when both oxygen and NO species are present. Moreover, our data indicate that NO-mediated oxidation is the predominant form of PTEN in aging brains and in MCI/AD brains (Figure 1D), which may generalize to other neurodegenerative diseases such as PD.

Although our mutagenesis studies (Figure 6B) cannot determine the nitrosylated sites unambiguously, the results strongly suggest that C83 is likely the most significant physiological site of S-nitrosylation on PTEN. It is possible that multiple Cys residues are involved depending on the spatial and temporal concentrations of NO. It is well known that the C124 residue is critical for the enzymatic activity of PTEN [32]; mutation of this residue results in inactivation of both the protein and phospholipid lipase activities of PTEN. Our data [see additional file 3] also indicate its importance in PTEN protein stability; mutation on this Cys residue renders PTEN resistance to SNOC-induced protein degradation. It warrants further investigation of the underlying mechanisms. Structural analysis based on the solved crystal structure of PTEN [33] indicates that Cys83 is not in close vicinity to C124, which is located face-to-face to C71 (Figure 6D). It is therefore not yet clear mechanistically how C83, in conjunction with C71 and C124, affects the lipid phosphatase activity of PTEN.

Our results demonstrate NO-mediated PTEN protein degradation via UPS, as evidenced by enhanced ubiquitination. There are several precedents showing that protein S-nitrosylation can be functionally coupled to its ubiquitination and modulate protein degradation [34-38]. It is possible that S-nitrosylation of PTEN plays a direct causative role in its degradation, as evidenced by enhanced ubiquitination upon SNOC treatment (Figure 4A). Given the role of phosphorylation in modulating PTEN protein stability and activity previously revealed by cancer cell models [14,38], it is also possible that NO signal induces alteration on PTEN phosphorylation status which is the cause of enhanced ubiquitination and protein degradation. Interestingly, the two putative kinases identified as responsible for phosphorylation of the two major clusters on PTEN (Ser/Thr cluster 380/382/383 and Thr366/Ser370), namely glycogen synthase kinase GSK3β and casein kinase CK2, are both implicated in neurodegeneration [39,40]. Moreover, our unpublished data show that OA can prevent PTEN dephosphorylation to a significant extent following exposure to SNOC, suggesting that PP2A, PP1 and perhaps PP2B, all major protein phosphatases implicated in AD [41], may play a role in modulating PTEN stability. Thus, the detailed interplay between kinases and phosphatases warrants further investigation.

Although we made our initial observation in MCI/AD brain samples, the loss of PTEN is also reported to occur in both myocardial [27] and cerebral ischemia/reperfusion [28,29]. Therefore, we believe that PTEN inhibition in these acute conditions mediates subsequent activation of PI3K/Akt signaling, which is known to be key to the endogenous protective effect, similar to the neuronal adaptive response. Paradoxically, we found that loss of PTEN occurs in human brains with AD, accompanied by elevated P-Akt in AD-affected regions. Moreover, the elevated P-Akt has often been detected in the same neuron bearing neurofibrillary tangles, which is a major pathological hallmark of AD consisting of hyperphosphorylated tau protein [13]. Although a reduction of P-Akt was also reported by previous studies [42]; we repeatedly found elevated P-Akt levels in degenerating neurons in AD brains by immunohistochemistry (unpublished data), which is consistent with several other reports [11,43]. Taken with our earlier finding that downregulated PTEN resulted in tau hyperphosphorylation and aggregation, we speculate that the loss of PTEN may be a contributing factor to neurodegeneration over the course of the disease due to chronically or excessively activated Akt signaling, which we speculate to be detrimental, as has been reported in several other chronic diseases [44,45]. Akt is activated in samples from patients with chronic heart failure; biochemical analyses demonstrated that chronic Akt activation induces feedback inhibition [46]. This nascent theory appears to be supported by a finding that conditional PTEN ablation in the forebrain region which caused impaired synaptic structure and function with concomitant constitutive activation of Akt and mTOR signaling [47]. An alternative view would be that the increase in Akt signaling occurs in response to damage, as an adaptive response, but fails to reach significantly protective levels under these chronic conditions. Only future experiments will be able to distinguish between these alternatives.

## Conclusions

In summary, our studies have demonstrated that S-nitrosylation of PTEN plays a major role in PTEN regulation in neuronal systems and that NO signaling induces S-nitrosylation and ubiquitination to modulate both PTEN protein degradation and enzymatic activity, which may represent the underlying regulatory mechanism of the PI3K/Akt signaling pathway in both acute and chronic neurodegeneration. A fuller understanding of the molecular mechanisms underlying the cascade of events leading to PTEN inhibition in both acute and chronic settings will advance our knowledge of PTEN regulation in the CNS. It may also be instrumental in future therapeutic design of novel target-based intervention in treating neurodegenerative diseases and perhaps other age-related conditions such as cancers, diabetes, cardiovascular ischemia and stroke.

## Methods

### Cell culture, treatment of neurotoxic reagents and transfection

Primary cultured cortical neurons (PRCN) and neuroblastoma N2a cells were prepared as described [48]. For the majority of experiments, freshly prepared SNOC (Sigma-Aldrich, St. Louis, MO) was added at 200 μM to 2-week-old cultured neurons for 30 min. SNOC was freshly prepared as described [49]. In brief, to prepare a 100 mM stock solution, 0.0069 g sodium nitrite and 0.0121 g l-cysteine were added to 950 μl H_2_O. Then, 50 μl of 10 N HCl is added to adjust pH to be 7.4. For glutamate (Sigma-Aldrich), Aβ_25-35 _peptides (Bachem, Torrance, CA), and staurosporine/STS (Sigma-Aldrich), neurons were treated for 4 hours before cell lysates were prepared. For suppression of NOS activity, l-NMMA (Sigma-Aldrich, 1 mM) was applied to neurons before exposure to neurotoxic reagents.

Transient transfections were performed with plasmid constructs for pEF-PTEN-WT (kindly provided by Dr. Hong Wu, UCLA, CA) and its site-directed mutagenized constructs (Cysteine to Alanine substitution).

### Human patient brains

Human brain samples were provided by UCSD, San Diego, CA and were analyzed with institutional permission under California and National Institutes of Health guidelines. Informed consent was obtained following the procedures of the Institutional Review Boards of the Sanford-Burnham Institute for Medical Research.

### Biotin-switch assay for detection of PTEN S-nitrosylation

A biotin switch assay for detection of SNO-PTEN was performed as previously described with minor modification [50]. PRCN cultures were exposed to various concentrations (10, 50, 100, 200 and 300 μM) of SNOC and old SNOC for 30 min, glutamate, STS, or Aβ_25-35 _for 4 h.

### Fluorometric measurement of S-nitrosylation of PTEN

S-nitrosylation of PTEN was measured as previously described [25].

### Detection of H_2_O_2_-mediated oxidation-band shift assay

Cells were lysed in buffer containing 2% SDS and 40 mM N-ethylmaleimide and 20 μg of protein was subjected to 10% SDS-PAGE under non-reducing condition as described [22,23].

### PTEN phosphatase assay-Malachite green assay

Recombinant PTEN, which was expressed in baculovirus, was assessed for the phosphatase activity as previously described [2]. Enzymatic activity of PTEN was quantified as activity measured relative to the control.

### Immunoprecipitation and Western blot analysis

IP-Western experiments were performed as described [48]. The following antibodies (Abs) were used: rabbit anti-PTEN Ab (Cell Signaling, Danvers, MA, USA); goat anti-PTEN (Santa Cruz, Santa Cruz, CA); mouse anti-α-tubulin Ab (Sigma, St. Louis, MO, USA); rabbit anti-P-Akt Ab (Cell Signaling, Danvers, MA, USA); rabbit Akt 1/2/3 (Santa Cruz, Santa Cruz, CA); mouse anti-mono-poly-Ub Ab (Enzo, Plymouth Meeting, PA); rabbit anti-NEDD4 Ab (Upstate, Charlottesville. VA, USA); mouse anti-β-actin Ab (Sigma); anti-mouse IgG and anti-rabbit IgG horseradish peroxidase-conjugated Abs (Chemicon, Temecula, CA, USA).

### Cycloheximide chase assay

PRCN cells were incubated with 50 μg/ml cycloheximide (Sigma, St. Louis, MO, USA) for the indicated times in the presence or absence of glutamate (200 μM). Cells were simultaneously treated with glutamate and cycloheximide. To examine whether the UPS is involved in nitrosative stress induced PTEN reduction, cells were co-treated with glutamate and 25 μM MG132. Cells lysates were then prepared for Western blot analysis.

### Sindbis virus-delivered RNA interference mediated silencing of PTEN in PRCN

Three different sets of siRNAs were designed by Ambion for rat PTEN (RefSeq NM_031606, Ambion). The annealed oligonucleotides encoding siRNAs were cloned into the pIRES-enhanced RFP (Invitrogen) using EcoRI/BamHI sites and the resulting siRNA-IRES-RFP fragment was further cloned into pSin-Rep5 (Invitrogen). The efficiency and specificity of PTEN-siRNAs were then assessed by RT-PCR and western blot analysis. Virus particles were generated and infection performed in primary neurons according to the manufacturer's protocol as described previously [13].

### Site-directed mutagenesis of PTEN

PTEN mutants were created by site-directed mutagenesis (QuikChange^® ^II Site-Directed Mutagenesis Kits, Stratagene, La Jolla USA) at C71, C83, C105, C124, C136, C211, C218, C250, C296, C304, and its double/triple mutants with various combinations. The primers for C71A forward 5'-TTTAAAGCATAAAAACCATTACAAGATATACAA-3' and reverse 5'-GTCATAATGTCTAGCAGCAAGATTGTATAT-3'; C83A forward 5'-GACACCGCCAAATTTAATGCCAGAGTTGCACAA-3' and reverse 5'-GATATTGTGCAACTCTGGCATTAATGGCGG-3'; C105A forward 5'-GAACTTATCAAACCCTTTGCTGAAGATCTTGAC-3' and reverse 5'-CCATTGGTCAAGATCTTCAGCAAAGGGTTTGAT-3'; C124A forward 5'-CATGTTGCAATTCACGCTAAAGCTGGAAAG-3' and reverse 5'-GTCCCTTTCCAGCTTTGCGTGAATTGCTGGAATTGCTGCAA-3'; C136A forward 5'-GACGAACTGGTGTAATGATAGCCGCATATTTAT-3' and reverse 5'-CCCGATGATATAAATATGCGGCTATCATTACAC-3'; C211A forward 5'-GTTCAGTGGCGGAACTGCCAATCCTCAGTTTG-3' and reverse 5'-CACAAACTGAGGATTGGCAGTTCCGCCACTGAA-3'; C218A forward 5'-CCTCAGTTTGTGGTCGCCCAGCTAAAGGTGAA-3' and reverse 5'-CTTCACCTTTAGCTGGGCGACCACAAACTGAGC-3'; C250A forward 5'-CAGCCGTTACCTGTGGCTGGTGATATCAAAG-3' and reverse 5'-CTTTGATATCACCAGCCACAGGTAACGGCTG-3'; C296A forward 5'-TCAGAAAAAGTAGAAAATGGAAGTCTAGCTGAT-3' and reverse 5'-CAAATGCTATGGATTTCTTGATCAGCTAGACTT-3'; C304A forward 5'- CAAGAAATCAGCATTGCCAGTATAGAGCGT-3' and reverse 5'-CTGCACGCTCTATACTGGCAATGCTATCGATTT-3' were utilized for PCR amplification, respectively. PCR amplification was performed according to the company's protocol. Once single mutants were constructed, we used these mutants as a template for further construction of double/triple mutants.

### Immunocytochemistry for neuronal morphology/dendritic structure and cell death (TUNEL) assays

IHC on PTEN (1:500) and MAP-2/NeuN (1:500/each) and apoptotic staining were performed as described [48] on two week-cultured neurons seeded on glass cover slips. In PTEN heterozygous mice were genotyped as described [51].

### Statistics

All quantitative data were presented as means ± SDV. Comparison between groups were analyzed with unpaired ANOVA using Graphpad PRIZM software (La Jolla, CA, USA) and values of *p *< 0.05 were considered to be significant.

## Competing interests

The authors declare that they have no competing interests.

## Authors' contributions

Author contributions: FFL designed research; Y-DK, TM, S-YD, XZ, Y-MC, and JS performed experiments; SAL, EM, HX, analyzed data. FFL and Y-DK wrote the paper. All authors have read and approved the final manuscript.

## Supplementary Material

Additional file 1 **The table of the patient brain information**. Click here for file

Additional file 2 **Additional data on the Cys mutants**. SNO-PTEN levels were determined by biotin-switch assays and data were analyzed by densitometry of the SNO-PTEN/total PTEN ratio, indicating that the majority of C211-304 located in the C2 domain do not seem to be the direct sites of S-nitrosylation. Click here for file

Additional file 3 **Effect of double and triple Cys mutants on PTEN protein stability upon SNOC treatment**. Various mutant plasmids were transiently introduced to N2a cells along with WT control and cells were treated with SNOC (200 μM) 48 h after transfection. The steady-state levels of PTEN were determined at various time points by Western blot analysis using anti-HA antibody to probe on the exogenously expressed PTEN. Click here for file
