# Supplementary figures and images for: NO signaling and S-nitrosylation regulate PTEN inhibition in neurodegeneration
Source: Mol Neurodegener. 2010 Nov 10;5:49. doi: 10.1186/1750-1326-5-49 (PMC2992530; doi:10.1186/1750-1326-5-49)

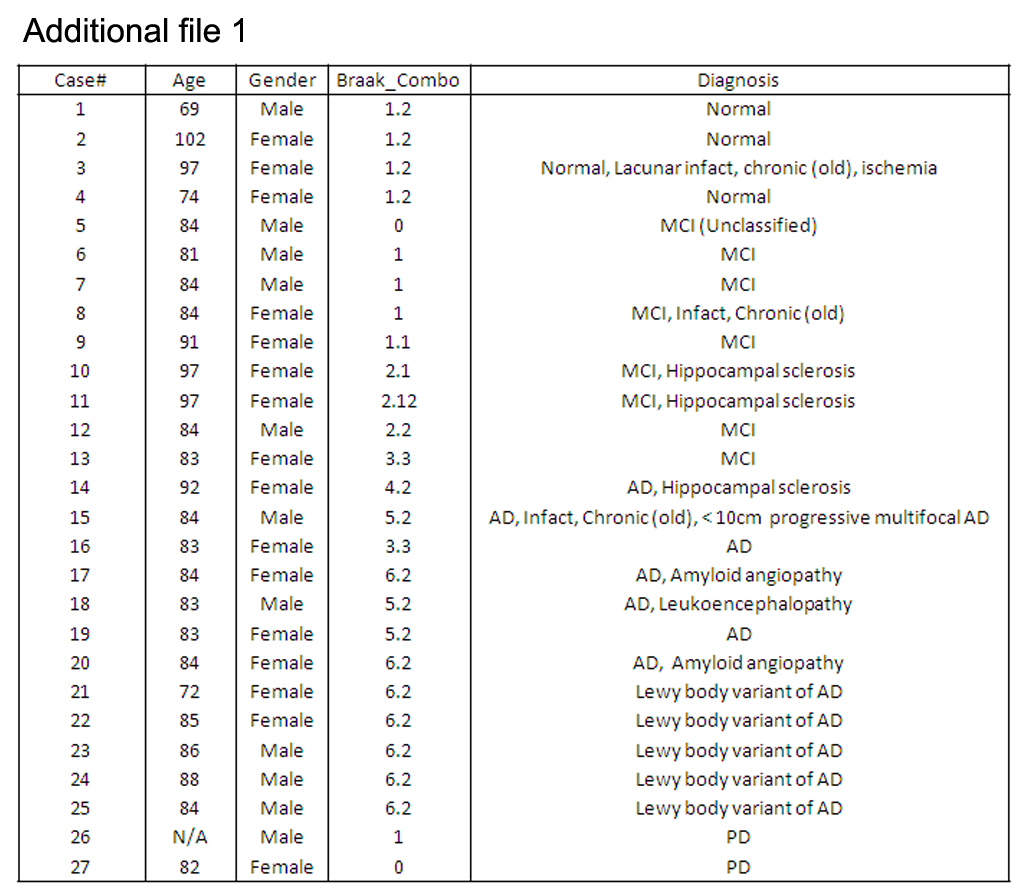

Supplement: Additional file 1 — The table of the patient brain information. [file 1750-1326-5-49-S1.TIFF]

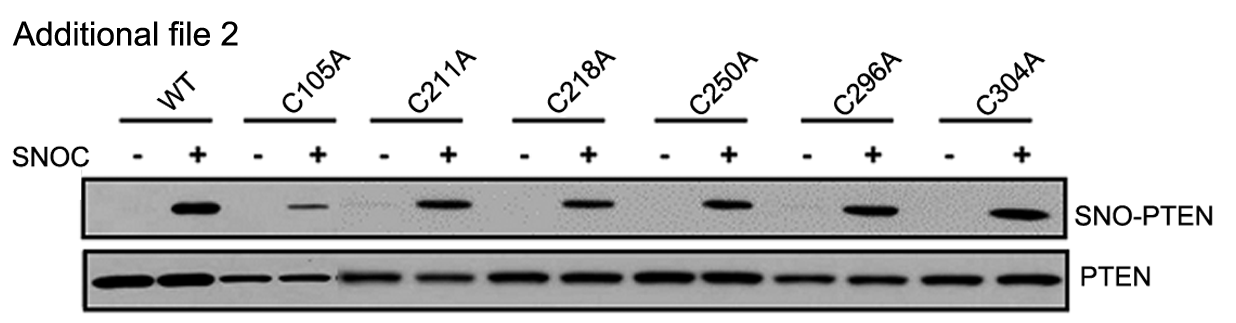

Supplement: Additional file 2 — Additional data on the Cys mutants. SNO-PTEN levels were determined by biotin-switch assays and data were analyzed by densitometry of the SNO-PTEN/total PTEN ratio, indicating that the majority of C211-304 located in the C2 domain do not seem to be the direct sites of S-nitrosylation. [file 1750-1326-5-49-S2.TIFF]

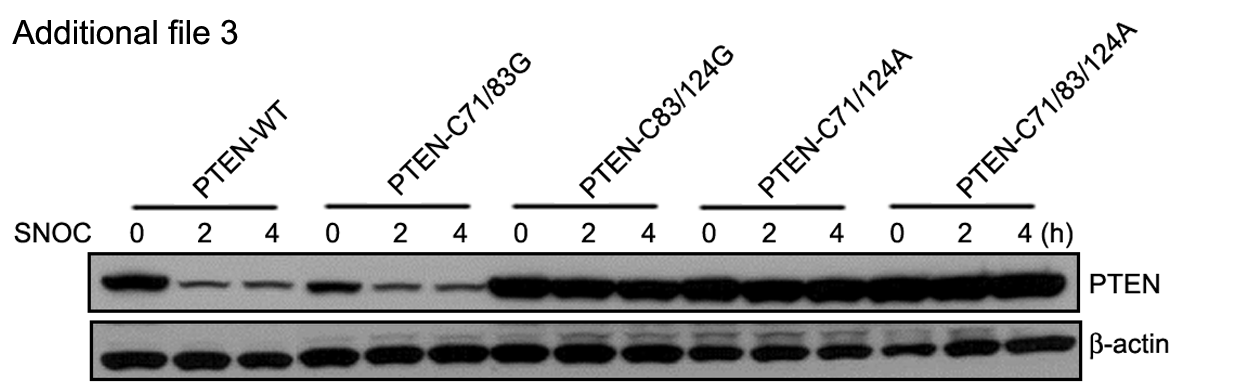

Supplement: Additional file 3 — Effect of double and triple Cys mutants on PTEN protein stability upon SNOC treatment. Various mutant plasmids were transiently introduced to N2a cells along with WT control and cells were treated with SNOC (200 μM) 48 h after transfection. The steady-state levels of PTEN were determined at various time points by Western blot analysis using anti-HA antibody to probe on the exogenously expressed PTEN. [file 1750-1326-5-49-S3.TIFF]
